# Analysis of the Relationship Between Psychological Well-Being and Decision Making in Adolescent Students

**DOI:** 10.3389/fpsyg.2020.01195

**Published:** 2020-07-10

**Authors:** Javier Páez-Gallego, José Alberto Gallardo-López, Fernando López-Noguero, María Pilar Rodrigo-Moriche

**Affiliations:** ^1^Department of Psychology, Universidad Europea de Madrid, Madrid, Spain; ^2^Department of Education and Social Psychology, Universidad Pablo de Olavide, Seville, Spain; ^3^Department of Pedagogy, Universidad Autónoma de Madrid, Madrid, Spain

**Keywords:** psychological well-being, decision-making, adolescents, gender, adaptive strategies, maladaptive strategies

## Abstract

Adolescents are frequently faced with situations in which they have to make decisions by choosing from a range of possible alternatives. In such circumstances, individual, social, and environmental conditions have an impact on the choice of the final decision in light of the various options presented. The main objective of this study is to analyze the relationship between the psychological well-being of adolescent students and their decision-making style. The research method used corresponds to an ex post facto, quantitative, transversal, correlational, and descriptive design, with an initial sample of 1,262 students from the Autonomous Community of Madrid, Spain, aged 13–19. A subsequent resampling of 385 participants was extracted from the initial sample by proportional allocation to strata (according to the levels of the variables gender, academic year, and educational institution classification) to guarantee the representativeness of the population data. Data collection uses the first Spanish adaptation of Ryff's Psychological well-being Scale and the Flinders Adolescent Decision Making Questionnaire, adapted by Friedman and Mann. The data shows that greater use of adaptive decision-making strategies correlates significantly with greater psychological well-being. In contrast, the correlation is high and negative at the intersection of the maladaptive decision-making variables and psychological well-being.

## Introduction

Psychological well-being is currently regarded as a construct which lacks a well-defined theoretical structure (González Barrón et al., 2002; Freire et al., 2017; Carneiro et al., 2019). However, the concept can be directly associated with how individuals assess their quality of life, and whether they give it a positive and favorable evaluation from a systemic and holistic perspective. As a consequence, psychological well-being can be related to high self-esteem, a positive disposition, and low depressive symptoms (Eronen and Nurmi, 1999).

From an evolutionary perspective, psychological well-being has been associated with the terms quality of life and mental health (Popescu, 2016; Loera-Malvaez et al., 2017; Sarafraz et al., 2019). In this respect, quality of life is understood from a multidimensional perspective that addresses the most relevant dimensions of an individual's life (Cancino et al., 2016; Losada-Puente, 2018). This includes material and non-material aspects, as described in Maslow's hierarchy of needs -physiological, safety, love, and belonging to social groups. However, mental health concerning psychological well-being has only ever been associated with non-material factors with a distinct clinical interpretation. Some of these factors are the creation of affective relationships with significant others, and the nurturing and development of self-esteem, self-concept or self-image (Latief and Retnowati, 2019).

The concept of psychological well-being takes into account the personal and social dimensions which individuals assess subjectively. Thus, many authors coincide in including questions relating to the field of social and emotional relationships (Rosa-Rodríguez et al., 2015; Latipun et al., 2019), as well as aspects connecting to family and work context (Mafud, 2016; Millán et al., 2017; Soto and Almagiá, 2017).

Although psychological well-being is usually understood as a personal endeavor to continually improve oneself, with the clear objective of self-realization in positive terms (Ballesteros et al., 2006), it should be noted that another notion also exists. The subjective assessment of well-being by individuals should be understood as the perceived absence of problems and/or the presence of pleasant and satisfying sensations (Villar et al., 2003; Freedman et al., 2017; Raleig et al., 2019). The above conceptualizations inherit the classic components of subjective well-being, which emphasize satisfaction with one's own life, development of capacities, and self-realization.

However, Ryff (1989) proposed a model that has acquired special relevance among the scientific community by bringing together the aspects from of all the previous conceptualizations. This model recognizes the subjective nature of psychological well-being, according to which individuals evaluate the variable according to their level of satisfaction with the six dimensions that make up the model (Díaz et al., 2006). These six dimensions -*self-acceptance, positive relations with others, environmental mastery, autonomy, purpose in life*, and *personal growth*- measure the subjective self-perception of social and family relationships, the achievement of personal and professional goals and to what extent they affect happiness, and the later perception of satisfaction with the personal and professional goals achieved (Uribe Urzola et al., 2018). Table 1 shows the content of each of the dimensions as used in this study, covering the whole spectrum of Ryff's definition the psychological well-being variable.

**Table 1:** Description of the dimensions proposed by Ryff and Keyes (1995).

**Dimension**	**Content**
Self-acceptance	An individual's positive or negative assessment of themselves, and their level of satisfaction with their self-concept. It implies the recognition of one's own strengths and weaknesses.
Positive relations with others	The ability to establish stable and satisfying long-term social relationships.
Environmental mastery	The ability to generate and choose favorable environments consistent with their and others' personal interests and tastes, and the ability to influence the environment positively.
Autonomy	An individual's ability to maintain their individuality in a variety of social contexts.
Purpose in life	An individual's ability to set long-term personal goals and establish ways to achieve them.
Personal growth	An individual's ability to implement strategies to development their potential to the full.

*Source:the authors based on Véliz Burgos (2012)*.

Adolescence is an evolutionary period in an individual's life in which all the variables of Ryff's psychological well-being are developed, put into play, and contrasted socially and individually. Consequently, from an international perspective, there are numerous studies that support the importance of evaluating psychological well-being during adolescence (Cotini de González et al., 2003; Figueroa et al., 2005; Romero et al., 2007; Balcázar Nava et al., 2008; Medina and Velásquez, 2017).

In this respect, the way adolescents value their peers and the power of the group is reflected in the assessments they make of the dimensions relating to the emotional ties they establish with their family, their peer group, and their own self-acceptance. There is an irrefutable relationship between psychological well-being and the variables of self-concept and quality of social relationships established by adolescents with family, partners and friends (Loera-Malvaez et al., 2017). Consequently, it is of interest to consider this particular moment of maturation and take into account the diversity of interests and that can be found in this specific stage in life.

As individuals, the ability to make decisions throughout our whole lives must be developed. However, this is closely related to cognitive maturation, the development of abstract thinking, and environmental mastery (Raleig et al., 2019). Consequently, adolescence is a fundamental stage in the development of decision-making capacity (Bosch et al., 2016; Modecki et al., 2017). Many important decisions are made during this development stage in different contexts including education, the family, and the peer group.

Decision-making has been identified as one of the main life skills which has a direct impact on psychological well-being and is defined as the ability to “take responsibility for one's own decisions, taking into account ethical, social and security aspects” (Bisquerra Alzina and Pérez Escoda, 2012, p. 73). To know the development of this competence, the appropriate use of problem solving strategies and the capacity for critical self-reflection and rational judgment are evaluated (Mieles and Alvarado, 2012).

Currently, different styles of decision making can be identified. Some of them are directly related to management and leadership models in the field of business management and organization. In this sense, styles such as managerial, conceptual, consultative, or consensus are identified (Cuadrado, 2015). However, these decision-making styles have not been taken into account in this research because they are far from the focus of our study.

From a historical perspective on the theoretical development of the term, Janis and Mann (1977) argue that decision-making falls into four different styles: vigilance, hypervigilance, avoidance, and complacency. The authors identified that there is always a period of conflict between the different alternatives and the values implicit in each one before making any decision. Each of these styles is characterized by their respective attributes or features:

- Vigilance: making decisions based on the systematic search and careful consideration of all viable alternatives in an unhurried, non-impulsive way with the aim of satisfying personal objectives.
- Hypervigilance: an impulsive, disorganized decision-making style which leads to feelings of insecurity and panic. This style disrupts the thought process inhibiting the correct assessment of the alternatives and their consequences.
- Avoidance: delegating decision-making to a third person, thereby ignoring the problem in hand.
- Complacency: unthinking adherence or change to simple courses of action; going along with what others say.

In turn, Janis and Mann (1977) maintain that everyone uses the four decision-making styles at some time or another, but that an individual's predominate style will depend on the frequency with which each one is used (Bernal et al., 2012). However, they also argue that vigilance exhibits optimal qualities for the adequate development of decision-making skills in adolescents, given that it is an adaptive style that leads to correct decisions.

A later trend in the study of decision-making was to process individuals' data as one of the key elements in the assessment process. As a result, Ross (1981) proposed a series of characteristics that an individual ought to possess in order to minimize the risk of failure when making decisions. These characteristics are: having the ability to identify many alternatives; establishing assessment criteria to reflect on the possible alternatives and assess them consistently; collecting and analyzing data on each alternative, and having the capacity for self-assessment (Ross, op. cit).

Taking the whole theoretical corpus into account, and focusing on the decision-making process by adolescents, Byrnes (2002) argues that there are four stages that must be followed in order to be considered competent. In this respect, the first stage would be to establish the desired objectives, then compile possible alternatives to meet the proposed objectives, prioritize the alternatives under criteria of importance and, finally, select the best alternative. However, it should be noted that an alternative is only appropriate according to the situational variables to which it responds. Hence, the ability to assess the context to which the decision-making action corresponds becomes essential. Thus, according to Gambara and González (2003), the decision-making context is relevant in determining the use of decision-making styles.

The following works below have performed studies to assess the development of decision-making skills, decision-making coping styles, and related psychological factors in adolescents. Some of the most important include Weithorn and Campbell's (1982) analysis aimed at observing the level of decision-making skills in children and adolescents of different ages with the aim of observing developmental differences. They found that 14 and 18-year olds obtained similar levels of decision-making skills.

In turn, other authors, such as Mann and Friedman (2002), identified that the minimum age for the development of decision-making competence is 15 and state that frequent use of the vigilance style may respond to the tendency for socially accepted responses. However, they also found that such responses had little to do with actual behavior.

Thus, Gambara and González (2003) observed that there are differences in decision-making skills by age. The older the adolescents are, the more effective their skills. The authors argue that knowledge obtained during adolescence, together with the particular characteristics of this evolutionary stage, does not increase the use of the vigilance decision-making style, but it rather causes the use of the other three styles to decrease considerably. In other words, younger adolescents use the maladaptive style more frequently.

Thus, as adolescents get older, they use the vigilance style more times, the only style that falls into the adaptive category. The data obtained from the Gambara and González study (op cit.) reveals that younger adolescents make more use of maladaptive styles. In relation to this, the conclusions suggest that adequate training in decision-making is synonymous with the appropriate development of decision-making skills. Similarly, statistically significant differences appear in the interaction between decision-making styles, where the vigilance style tends to dominate.

Bethencourt and Cabrera (2011) highlight the variable self-esteem as correlating with the use of different decision-making styles. In this respect, high self-esteem generates greater confidence in and commitment to the decisions made, which is consistent with the vigilance style. However, low self-esteem may be associated with less confident decision making which leads to styles that resonate more with complacency and avoidance. This, in turn, gives rise to a series of consequences including distorting the view of the situation, which may lead to incorrect decisions (Di Fabio and Blustein, 2010). In the same vein, Cascio et al. (2016) highlight the role of parents in the development of adolescents' self-esteem and its correlation in decision-making performance. In this respect, their study observed that when families perform parenting styles based on confidence in their children's abilities, the children develop firm and secure self-esteem that allows them to make rational decisions. Furthermore, these results can be observed in a similar way in both genders.

Equally, the study performed by Moreno et al. (2011) shows a significant relationship between self-esteem, self-efficacy, self-ability, and self-efficiency with different decision-making styles, depending on the age of the participants. In turn, Carbia et al. (2017) highlight the same variables and coping strategies to explain the differences between males and females. Thus, differences were not found between the genders in the final results obtained but in the decision-making processes themselves.

By the same token, some authors have applied the study of decision-making in adolescents to the vocational sphere, differentiating between males and females (Abidin et al., 2019; Hechtlinger et al., 2019; Kvasková and Almenara, 2019). In these studies, differences were found in the decision-making process variables by gender; the female sample obtained scores better than the male sample.

Other studies, such as those by Gil et al. (2010) include variables such as time and anxiety in the decision-making process. Their findings indicate a remarkable increase in the use of the hypervigilance style in all age groups, although older adolescents primarily continue to use the vigilance style.

However, it can be observed that the different psychological and situational variables of adolescence directly correlate with the decision-making capacity and decision-making style employed. In addition, the results found in the above studies show differences by age and gender. Among all the variables, gender is particularly relevant. As such, one of our main objectives is to study the differences in the use of decision-making styles based on the values given by gender.

There are previous studies to take into account that analyze decision-making and social welfare (Yellen and Cella, 1995; Smerglia and Deimling, 1997; Rudd et al., 2012; Rutledge et al., 2015) as well as other more contemporary studies that address the impact of social relationships and the regulation of emotions on decision making (Wong et al., 2019; You et al., 2019). It is also important to investigate the cognitive processes involved in the development of decision-making skills (Jin et al., 2019; Yilmaz and Kafadar, 2019), the stages of the decision-making process, and the constraints that affect its development (Lucks et al., 2020).

## Materials and Methods

The aim of this study is to analyze if there is a relationship between the psychological well-being of adolescent students and their decision-making style by age and gender.

The research method used responds to an ex post facto, quantitative, transversal, correlational, and descriptive design, with an initial sample of 1,262 students from nine centers of Compulsory Secondary Education and High Schools in the Autonomous Region of Madrid (Spain) aged 13–19 (x = 15.80); σ = 1.714), and a subsequent re-sampling of 385 participants, extracted from the initial sample by proportional allocation to strata (according to the levels of the variables gender, academic year, and center ownership) to guarantee the representativeness of the population data. The final sample comprised 206 male (53.5%) and 179 female (46.5%) students.

The variables in the methodological design of the study were: sociodemography, psychological well-being, and decision-making styles.

Two methods were used for data collection:

- First, the Ryff Psychological Well-being Scale (1989) adapted by van Dierendonck (2004) and translated into Spanish by Díaz et al. (2006). This scale collects data on the variable psychological well-being, based on the subjective assessment by adolescents of different situations and questions relating to their living situation, and their perception of success in everyday aspects of development and achievement, taking into account the six dimensions of the model described in Table 1: *self-acceptance, positive relations with others, environmental mastery, autonomy, purpose in life*, and *personal growth*. The scale comprises a total of 39 items in a Likert-type format with values in a range of 1 to 6, where 1 is totally disagree and 6 is totally agree. Cronbach's alpha is used to measure the internal consistency of the psychological well-being subscales as follows: *self-acceptance* (0.83), *positive relations with others* (0.81), *environmental mastery* (0.71), *autonomy* (0.73), *purpose in life* (0.83) and *personal growth* (0.68).
- Second, a questionnaire on decision-making styles -Flinders Adolescent Decision Making Questionnaire (FADMQ)- adapted by Friedman and Mann (1993). Decision-making styles are understood as the general trend in the use of strategies aimed at reaching a solution to a problem posed. In this sense, this questionnaire measures two major decision-making styles –adaptive and maladaptive. The former is the most optimal and desirable in the decision-making process as it responds to criteria of rationality in the evaluation of alternative solutions and their consequences. In contrast, the maladaptive style is not considered optimal as it is mediated by biases and heuristics. Thus, this method is based on the normative models of decision-making, which emphasize the rational component of the decision-making process. In addition, Dawes and Hastie (2010) argue that control model must be based on tangible elements in order to be considered rational, i.e., have a direct impact on individuals' physiological and/or psychological well-being, psychological skills and socio-emotional attributes.

The reliability study of both subscales of the model gave rise to high values of Cronbach's alpha reliability coefficient. Thus, the internal consistency of the five items on the maladaptive subscale is 0.74 and the internal consistency of the seven items on the adaptive subscale is 0.78.

Finally, the Statistical Package for the Social Sciences (SPSS V26) software was used to perform descriptive, factorial, correlational, mean contrast, and variance analyses.

## Results

### Psychological Well-Being

The scores obtained by the sample subjects on the Ryff (1989) psychological well-being scale show an average of over 140 points (= 147.06). Considering that the range of scores on this scale varies between 39 and 234 points, this statistic places the sample slightly above the mean score (137 points). This puts the sample subjects within the category of average psychological well-being according to the interpretation of the scores on the scale itself.

In addition, the standard deviation shows a moderate dispersion (ó = 17.807).

In turn, the means obtained for the six factors that comprise the psychological well-being variable range from 4.18 (= 4.18) to 4.59 (= 4.59) as seen in Table 2 with small dispersions between 0.606 and 0.825.

**Table 2:** Mean scores and dispersion in psychological well-being and dimensional factors.

		**Psychological well-being**	**Self-acceptance**	**Positive relation with others**	**Autonomy**	**Environmental mastery**	**Personal growth**	**Purpose in life**
Total	Mean	147.06	4.33	4.19	4.18	4.24	4.59	4.40
	Standard deviation	17.807	0.825	0.756	0.723	0.668	0.606	0.800
Males	Mean	147.49	4.45	4.17	4.19	4.25	4.54	4.43
	Standard deviation	16.708	0.741	0.720	0.667	0.646	0.624	0.756
Females	Mean	146.57	4.19	4.22	4.18	4.23	4.64	4.37
	Standard deviation	19.027	0.892	0.796	0.785	0.693	0.583	0.845

*Arithmetic mean; Standard deviation; N Total = 385, N Male= 206, N Female = 179*.

However, if mean scores are analyzed by gender, male psychological well-being data are slightly higher (= 147.49) than females (= 146.57).

In turn, both genders obtain very similar scores on the six variables that comprise psychological well-being (Table 2), with males outcome slightly higher in the factors of *self-acceptance, environmental mastery, autonomy*, and *purpose in life*. Females results are also higher in the factors of *positive relations with others* and *personal growth*. Nevertheless, the differences are minimal.

### Differences in the Psychological Well-Being of Adolescents by Gender

First, the sample homoscedasticity was analyzed, which obtained equality of variances in all the variables, except *autonomy*. Consequently, the results of the analysis of the mean difference that did not assume equality of variances are used (Table 3).

**Table 3:** Levene's test for equality of variances by gender and differences in psychological well-being and their factors by gender.

	**F**	**Sig**.	**t**	**gl**	**Sig. (bilat.)**
Psychological well-being	4.127	0.063			
Self-acceptance	2.326	0.128	0.590	383	0.556
Positive relation with others	0.818	0.366	3.227	383	0.001
Autonomy	5.335	0.021	−0.533	383	0.594
Environmental mastery	1.492	0.223	0.204	351.49	0.839
Personal growth	0.142	0.706	0.311	383	0.756
Purpose in life	3.289	0.071	−1.529	383	0.127

*Levene test; N = 385; Student t analysis, N = 385*.

Thus, an analysis for equality of mean for the *psychological well-being* variable by gender shows that there are statistically significant differences between the two samples in *self-acceptance* (t = 3.227; Sig. = 0.001) (Table 3).

As a result, using Cohen's *d* index to analyze the size of the effect, it can be observed that the differences are not significant.

### Decision-Making Style

As regards adolescent decision-making styles, scores on the scale measuring the maladaptive style range from 0 to 15. In turn, scores on the adaptive style range from 0 to 21. The average score for the sample subjects using both styles is shown in Table 4.

**Table 4:** Use of decision-making styles.

		**Mean**	**Standard deviation**
Maladaptive style	Total	9.34	2.694
	Males	9.41	2.628
	Females	9.26	2.773
Adaptive style	Total	20.26	3.259
	Males	20.70	3.034
	Females	19.74	3.437

*Arithmetic mean; Standard deviation; N Total = 385, N Male = 206, N Female = 179*.

The results show an above-average score on the subscale measuring the maladaptive style (mean = 9.34) and a very high score for the subscale measuring the adaptive style, where the sample scored almost the maximum number of 21 points (mean = 20.26).

In turn, if scores by gender are analyzed, it can be observed that the male sample scores slightly higher than the female sample in both styles. However, in both cases the scores are above average on the subscale for the maladaptive style and practically at the very top of the subscale for the adaptive decision-making style (Table 4).

Analyzing the use of decision-making styles based on the groups formed by the sex variable, different results can be observed (Table 5).

**Table 5:** Differences in decision-making styles by gender.

	**T**	**gl**	**Sig. (bilateral)**
Maladaptive style	0.527	383	0.598
Adaptive style	2.914	383	0.004

*Student t analysis; N = 385*.

On the one hand, both sexes use in the same way maladaptive decision-making strategies (*t* = 0.527; *p* = 0.598). On the contrary, there are statistically variable differences in the use of adaptive decision-making strategies, being used to a greater extent by the group of men (*t* = 2,914; *p* = 0.004).

### Psychological Well-Being and Adaptive Decision-Making Style

The correlation between the values given by the sample to psychological well-being and its factors show statistical significance in all combinations with the adaptive decision-making style (Table 6). Furthermore, the correlation is positive across the board, which shows that greater use of adaptive decision-making strategies is directly related to greater psychological well-being.

**Table 6:** Correlations between psychological well-being and the adaptive decision-making style.

			**Psychological well-being**	**Self-acceptance**	**Positive relation with others**	**Autonomy**	**Environmental mastery**	**Personal growth**	**Purpose in life**
Adaptive style	Total	Correlation	0.544^**^	0.485^**^	0.242^**^	0.359^**^	0.472^**^	0.346^**^	0.473^**^
		Sig. (bilat)	0.000	0.000	0.000	0.000	0.000	0.000	0.000
	Male	Correlation	0.463^**^	0.354^**^	0.201^**^	0.262^**^	0.396^**^	0.370^**^	0.414^**^
		Sig. (bilat)	0.000	0.000	0.004	0.000	0.000	0.000	0.000
	Female	Correlation	0.617^**^	0.574^**^	0.291^**^	0.448^**^	0.549^**^	0.358^**^	0.528^**^
		Sig. (bilat)	0.000	0.000	0.000	0.000	0.000	0.000	0.000

*Correlation coefficient; N Total = 385, N Male= 206, N Female = 179*.

** *The correlation is significant at 0.01 (bilateral)*.

Thus, the adaptive decision-making style correlates significantly with a probability of <0.01 with overall psychological well-being (*r* = 0.544; *p* = 0.000) and with *self-acceptance* (*r* = 0.485; *p* = 0.000), *positive relations with others* (*r* = 0.242; *p* = 0.000), *environmental mastery* (*r* = 0.472; *p* = 0.000), *autonomy* (*r* = 0.359; *p* = 0.000)*, purpose in life* (*r* = 0.473; *p* = 0.000), and *personal growth* (*r* = 0.346; *p* = 0.000) (Table 6).

In the male sample (*N* = 206), the relationship between the adaptive decision-making style and the psychological well-being variable (*r* = 0.463; *p* = 0.000), and its six factors shows a positive correlation with a probability of < 0.01 in the table below *total psychological well-being* (*r* = 0.463; *p* = 0.000)*, self-acceptance* (*r* = 0.354; *p* = 0.000), *positive relations with others* (*r* = 0.201; *p* = 0.004), *environmental mastery* (*r* = 0.396; *p* = 0.000), *autonomy* (*r* = 0.262; *p* = 0.000)*, purpose in life* (*r* = 0.414; *p* = 0.000), and *personal growth* (*r* = 0.370; *p* = 0.000) (Table 6).

In the female sample, the adaptive decision-making style shows a positive correlation with the psychological well-being variable (*r* = 0.617; *p* = 0.000). Similar results are obtained with the factors *self-acceptance* (*r* = 0.574; *p* = 0.000), *positive relations with others* (*r* = 0.291; *p* = 0.000), *environmental mastery* (*r* = 0.579; *p* = 0.000), *autonomy* (*r* = 0.448; *p* = 0.000)*, purpose in life* (*r* = 0.528; *p* = 0.000), and *personal growth* (*r* = 0.358; *p* = 0.000) (Table 6).

### Psychological Well-Being and Maladaptive Decision-Making Style

In contrast, the maladaptive decision-making style correlates negatively with a probability of <0.01 with overall psychological well-being (*r* = −0.458; *p* = 0.000) and with the factors *self-acceptance* (*r* = −0.323; *p* = 0.000), *positive relations with others* (*r* = −0.276; *p* = 0.000), *environmental mastery* (*r* = −0.357; *p* = 0.000), *autonomy* (*r* = −0.399; *p* = 0.000)*, purpose in life* (*r* = −0.278; *p* = 0.000), and *personal growth* (*r* = −0.348; *p* = 0.000) (Table 7).

**Table 7:** Correlations between psychological well-being and the maladaptive decision-making style.

			**Psychological well-being**	**Self-acceptance**	**Positive relation with others**	**Autonomy**	**Environmental mastery**	**Personal growth**	**Purpose in life**
Maladaptive style	Total	Correlation	−0.458^**^	−0.323^**^	−0.276^**^	−0.399^**^	−0.357^**^	−0.348^**^	−0.278^**^
		Sig. (bilat)	0.000	0.000	0.000	0.000	0.000	0.000	0.000
	Male	Correlation	−0.458^**^	−0.323^**^	−0.276^**^	−0.399^**^	−0.357^**^	−0.348^**^	−0.278^**^
		Sig. (bilat)	0.000	0.000	0.000	0.000	0.000	0.000	0.000
	Female	Correlation	−0.516^**^	−0.402^**^	−0.275^**^	−0.419^**^	−0.448^**^	−0.404^**^	−0.359^**^
		Sig. (bilat)	0.000	0.000	0.000	0.000	0.000	0.000	0.000

*Correlation coefficient; N Total = 385, N Male = 206, N Female = 179*.

** *The correlation is significant at 0.01 (bilateral)*.

In turn, in the male sample, the maladaptive style also obtains a statistically significant correlation, but with a negative value. In consequence, an inverse relationship between psychological well-being (*r* = −0.400; *p* = 0.000) and the use of the above-mentioned decision-making style is established. The same applies to s*elf-acceptance* (*r* = −0.256; *p* = 0.000) *positive relations with others* (*r* = −0.277; *p* = 0.000), *autonomy* (*r* = −0.380; *p* = 0.000), *environmental mastery* (*r* = −0.268; *p* = 0.000), *personal growth* (*r* = −0.300; *p* = 0.000), and *purpose in life* (*r* = −0.198; *p* = 0.000) (Table 7).

In turn, the correlational analysis of maladaptive decision-making style with psychological well-being shows a statistically significant negative correlation (*r* = −0.617; *p* = 0.000). Similarly, the use of the maladaptive style correlates negatively with *self-acceptance* (*r* = −0.574; *p* = 0.000) *positive relations with others* (*r* = −0.291; *p* = 0.000), *autonomy* (*r* = −0.448; *p* = 0.000)*, environmental mastery* (*r* = −0.549; *p* = 0.000), *personal growth* (*r* = −0.358; *p* = 0.000), and *purpose in life* (*r* = −0.528; *p* = 0.000) with a probability of <0.01 (Table 7).

The results of the correlational analyses of the total sample, as well as both gender samples, show a direct relationship between the use of adaptive decision-making strategies and psychological well-being, in which higher levels of psychological well-being correspond to a marked preference for using the adaptive decision-making style and vice versa.

In contrast, it was observed that the participants, both in the total sample and by gender, have an inverse relationship between the use of maladaptive decision-making strategies and psychological well-being. Thus, a more marked preference for the maladaptive decision-making style corresponds to a lower level of psychological well-being and its factors.

### Predictive Model for Adaptive Style in the Total Group

After checking the relationship between the variable Psychological Well-being and the Adaptive Decision-Making Style, the linear regression analysis is performed to build a model to predict the use of adaptive strategies based on psychological well-being and its dimensions.

The proportion of data in which it is possible to predict the use of adaptive strategies based on psychological well-being is 34.5% (R squared = 0.345) and the analysis of variance shows that the probability associated with the F statistic is lower to 0.05 (*F* = 33.201; *P* = 0.000).

In this way, the model is made up of the value of the constant (*C*= 6.132) and the coefficients with a probability <0.05 of the variables *Self-acceptance* (*C*_f_ = 152; *p* = 0.000), *Positive relations* (*C*_f_ = −0.071; *p* = 0.028), *Autonomy* (*C*_f_ = 0.098; *p* = 0.000), *Environmental mastery* (*C*_f_ = 0.112; *p* = 0.025), and *Purpose in life* (*C*_f_ = 0.143; *p* = 0.000), as shown in Table 8.

**Table 8:** Coefficients of the adaptive style variables in the total group.

	**Coefficient**	**Sig**.
Constant	6.132	0.000
Self-acceptance	0.152	0.000
Positive relation with others	−0.071	0.028
Autonomy	0.098	0.000
Environmental mastery	0.112	0.025
Personal growth	0.073	0.054
Purpose in life	0.143	0.000

The algorithm that explains the predictive model is:

*y* = *6.13* + *0.152x* + *0.098x* + *0.112x* + *0.143x* + *(* − *0.071x)*.

### Predictive Model for Maladaptive Style in the Total Group

Similarly, linear regression analysis is performed to build a model that allows predicting the use of maladaptive strategies based on psychological well-being and its dimensions.

The R squared value of the model shows that the proportion of data in which it is possible to predict the use of maladaptive strategies based on psychological well-being is 24.1% (R squared = 0.241).

On the other hand, the analysis of variance shows that the probability associated with the F statistic is <0.05 (*F* = 19,987; *P* = 0.000), being able to confirm the construction of a predictive model with these two variables.

With this, the model is formed by the value of the constant (*C* = 20,629) and the coefficients with a probability <0.05 of the variables *Autonomy* (*C*_f_ = −0.127; *p* = 0.000) and *Personal growth* (*C*_f_ = −0.119; *p* = 0.000), as shown in Table 9.

**Table 9:** Coefficients of the maladaptive style variables in the total group.

	**Coefficient**	**Sig**.
Constant	20.629	0.000
Self-acceptance	−0.024	0.490
Positive relation with others	−0.011	0.706
Autonomy	−0.127	0.000
Environmental mastery	−0.077	0.080
Personal growth	−0.119	0.000
Purpose in life	−0.012	0.717

Thus, the algorithm that explains the predictive model is

y = 20.63 + (−0.127x) + (−0.119x).

That is, the use of maladaptive decision-making strategies is equal to the constant plus the decrease in *Autonomy* by 0.127 times and the decrease in *Personal growth* by 0.119 times.

### Predictive Model for Adaptive Style in the Group of Men

The linear regression analysis for the construction of a model that allows predicting the use of adaptive strategies based on psychological well-being and its dimensions in the group of men shows that the proportion of data in which it is possible to predict the use of strategic dimensions is of 26.1% (R squared = 0.261) and the analysis of variance shows that the probability associated with the *F* statistic is < 0.05 (*F* = 11.711; *P* = 0.000).

In this way, the model is formed by the value of the constant (*C* = 7,554) and the coefficients with a probability of <0.05 for the variables *Personal growth* (*C*_f_ = 0.140; *p* = 0.005) and *Purpose in life* (*C*_f_ = 0.146; *p* = 0.005), as shown in Table 10.

**Table 10:** Coefficients of the adaptive style variables in the group of men.

	**Coefficient**	**Sig**.
Constant	7.554	0.000
Self-acceptance	0.065	0.265
Positive relation with others	−0.055	0.251
Autonomy	0.064	0.101
Environmental mastery	0.100	0.136
Personal growth	0.140	0.005
Purpose in life	0.146	0.005

The algorithm that explains the predictive model is

y = 7.55 + 140x + 146x.

### Predictive Model for Maladaptive Style in the Group of Men

On the contrary, the linear regression analysis for the construction of a model that allows predicting the use of maladaptive strategies based on psychological well-being and its dimensions in the group of men shows that the proportion of data in which it is possible to predict the use of adaptive strategies based on psychological well-being is 20% (R squared = 0.200) and the analysis of variance shows that the probability associated with the *F* statistic is <0.05 (*F* = 47.302; *P* = 0.000).

With this, the model is formed by the value of the constant (*C* = 19.826) and the coefficients with a probability <0.05 of the variables *Autonomy* (*C*_f_ = −0.143; *p* = 0.000) and *Personal growth* (*C*_f_ = −0.107; *p* = 0.016), as shown in Table 11.

**Table 11:** Coefficients of the maladaptive style variables in the group of men.

	**Coefficient**	**Sig**.
Constant	19.826	0.000
Self-acceptance	0.002	0.969
Positive relation with others	−0.045	0.291
Autonomy	−0.143	0.000
Environmental mastery	−0.027	0.648
Personal growth	−0.107	0.016
Purpose in life	−0.008	0.856

Thus, the algorithm that explains the predictive model is

y = 19.83 + (−0.143x) + (−0.107x).

That is, the use of maladaptive decision-making strategies is equal to the constant plus the decrease in *Autonomy* by 0.143 times and the decrease in *Personal growth* by 0.107 times.

### Predictive Model for Adaptive Style in the Group of Women

Regarding the group of women, the linear regression analysis of the adaptive style based on psychological well-being and its dimensions, shows that the proportion of data in which it is possible to predict the use of adaptive strategies based on psychological well-being is 44.2% (R squared = 0.442) and the analysis of variance shows a probability associated with the *F* statistic of <0.05 (*F* = 22.68; *P* = 0.000).

Therefore, according to the values shown in Table 12, the model is made up of the value of the constant (*C* = 5.314) and the coefficients with a probability <0.05 of the variables *Self-acceptance* (Cf = 0.176; *p* = 0.002), *Autonomy* (Cf = 0.130; *p* = 0.000) and *Purpose in life* (*C*_f_ = 0.150; *p* = 0.008).

**Table 12:** Coefficients of the variables of maladaptive style in the group of women.

	**Coefficient**	**Sig**.
Constant	5.314	0.003
Self-acceptance	0.176	0.002
Positive relation with others	−0.064	0.141
Autonomy	0.130	0.000
Environmental mastery	0.130	0.076
Personal growth	0.009	0.873
Purpose in life	0.150	0.008

The algorithm that explains the predictive model is

y = 5.31 + 176x + 130x + 150x.

### Predictive Model for Maladaptive Style in the Group of Women

Finally, the linear regression analysis of the variables Adaptive strategies in function of psychological well-being and its dimensions in the group of women, shows that the proportion of data in which it is possible to predict the use of adaptive strategies in function of the psychological well-being of the 30.2% (R squared = 0.302) and the analysis of variance shows that the probability associated with the *F* statistic is <0.05 (*F* = 12,399; *P* = 0.000).

In this way, the model is formed by the value of the constant (*C* = 21,457) and the coefficients with a probability <0.05 of the variables *Autonomy* (*C*_f_ = −0.109; *p* = 0.001), Environmental mastery (*C*_f_ = −0.133; *p* = 0.045), and *Personal growth* (*C*_f_ = −0.135; *p* = 0.011), as shown in Table 13.

**Table 13:** Coefficients of the adaptive style variables in the group of women.

	**Coefficient**	**Sig**.
Constant	21.457	0.000
Self-acceptance	−0.040	0.428
Positive relation with others	0.018	0.649
Autonomy	−0.109	0.001
Environmental mastery	−0.133	0.045
Personal growth	−0.135	0.011
Purpose in life	−0.012	0.816

The algorithm that explains the predictive model is

y = 21.46 + (−0.109x) + (−0.133x) + (−0.135x).

## Discussion

The objective of this study was to analyze the potential relationship between adolescents' psychological well-being and their decision-making styles, using Ryff's (1995) dimensions of psychological well-being, and Janis and Mann's (1977) decision-making model. Moreover, differences in the relationship by age and gender were also analyzed, which covers the current gap in this area of research according to gender.

### Phycological Well-Being

In reference to the level of psychological well-being, the adolescent students in the study scored slightly above the scale's average. Furthermore, this score is more than 30 points higher than the scores obtained in similar studies (Figueroa et al., 2005; Escarbajal et al., 2014).

Moreover, if present results are compared with other studies that analyze differences by gender, similar results are found, with psychological well-being slightly higher in males; although the differences are not significant (Zubieta et al., 2012; García, 2016).

Similarly, if the differences in terms of the dimensions that constitute the psychological well-being variable are observed, some studies point to high scores in either of the two gender.

Thus, for example, García (2016) concludes that females obtain significantly higher scores than males in the *personal growth* dimension. This result is similar to that found in our study, although in this case the difference is not significant.

In turn, the research performed by Zubieta et al. (2012) shows that males obtain higher scores than females in the *autonomy* dimension. However, females outperform males in all other dimensions—*positive relations with others, self-acceptance, environmental mastery, personal growth*, and *purpose in life*. These data contrast with those found in this study, in which males obtained higher scores than females in the *autonomy* dimension, but also in *self-acceptance, environmental mastery*, and *purpose in life*. However, none of the differences are significant.

Lastly, Raleig et al. (2019) highlight the higher score obtained by females in the *positive relations with other* dimension, which is similar to the difference found in the aforementioned results.

### Decision-Making Style

The values found regarding the use of decision-making styles show similar results to those of previous studies. Thus, Bosch et al. (2016) found the same preferential use of the adaptive style by adolescents, although they highlight some variables, such as anxiety or negative interpretation of ambiguous stimuli, that may have a negative impact on use.

However, this is an area that covers a wide field of development, given that there are no prior studies that analyze the preferred decision-making style by gender.

### Psychological Well-Being and Decision-Making Style

The results obtained in this study, concerning the relationship between psychological well-being and decision-making style, show a significant and positive correlation between adaptive decision-making strategies and all the psychological well-being variables. In contrast, the preferred use of the maladaptive decision-making style correlates both significantly and negatively with the psychological well-being factors. These results coincide with those found by Trujillo et al. (2015), who conclude that adolescents identify with improvised decision-making processes, the assumption being that this is justified by the desire to live in the moment and a sense of reward from quick-thinking, risky behavior. Similarly, the results obtained in their study reveal that adolescents have a strong perception of external loci of control on their lives.

However, the results obtained show a relationship between the psychological well-being of adolescents and their preferred decision-making style. This is in line with Pincham et al. (2019) hypotheses on adolescents at risk, which states that improving adolescents' psychological well-being would also mean improving their decision-making skills and the feedback process.

In this regard, it is important to call attention to the conceptualization of the term “decisional competence” according to (Bisquerra Alzina and Pérez Escoda, 2012), and the implications highlighted by Mieles and Alvarado (2012), who argue that the capacity of critical self-reflection and rational judgment is essential for the development of decisional competence. Moreover, these variables must be related to the factors of personal growth and purpose in life in Ryff's model of psychological well-being.

Lastly, Moreno et al. (2011) identified a relevant role for self-esteem, self-efficacy, self-capacity, and self-efficiency in the choice of decision-making strategies. They also found differences by age.

In turn, numerous studies have analyzed the differences between levels of psychological well-being (Ruck et al., 2014; To et al., 2017) and preferential decision-making styles by gender (Blustein and Phillips, 1990; Chaturvedi and Kumari, 2015; Camuñas, 2017; Pincham et al., 2019).

In this respect, the study performed by Trujillo et al. (2015) is particularly relevant. They identified a decision-making style they call “dependent” that has similarities with the maladaptive style outlined in our study which is associated with conditions of low self-esteem, difficulty in establishing stable affective relationships, and lack of purpose in life. These conditions are, in turn, related to low psychological well-being. According to the results, this dependent style is used equally by males and females. In contrast, connecting to the same study, the style defined as “rational” is associated with the careful selection of strategies aimed at achieving future objectives, a hypothesis that also argues that purpose in life and personal growth are essential factors in the psychological well-being variable. Again, these results are also found both in males and females.

These results are similar to those obtained in the present study; in both cases, subjects with a preferential use for adaptive decision-making strategies also have a high score in psychological well-being and in the factors that comprise the variable, and vice versa. However, in his study on decisional competence, Camuñas (2017) found differences in the variables by gender associated with factors such as anxiety or stress in sport. Similar results were found by Gil et al. (2010), who include time and anxiety as important variables in the preferential use of the hypervigilance style throughout adolescence.

## Conclusions

### Psychological Well-Being

The conclusions of the analyzes carried out in accordance with the objectives of the study, and in comparison with other studies, highlight the fact that adolescents assess their psychological well-being above the average of other profiles of adolescents analyzed. This aspect is directly linked with good quality of life and mental health.

The factors that explain and determine psychological well-being vary greatly depending on evolutionary characteristics and personality traits.

It is important to bear in mind that social phenomena directly affecting gender roles have a direct impact on factors such as establishing relationships, life goals, and behavior relating to autonomy. Thus, gender is a demographic variable that can have an important impact on psychological well-being and its factors.

The slight differences between males and females in the subjective perception of psychological well-being makes advisable to continue establishing educational programs that enhance the dimensions of *self-acceptance, environment mastery, autonomy*, and *purpose in life* in females; and develop *positive relationships with others* and *personal growth* in males. A compensatory approach to these differences would enhance the perception of subjective well-being in both genders.

### Decision-Making Style

The conclusions of this study suggest that adequate training in decision-making involves the appropriate development of decision-making skills. Similarly, there are statistically significant differences in the interaction between decision-making styles, with the adaptive style the preferred choice.

Differentiating by gender, males score higher than females in both adaptive and maladaptive decision-making styles. However, these differences are not statistically significant for the variable maladaptive style, and it can be concluded that both genders make similar use.

In contrast, the difference between genders for the adaptive style is statistically significant, which enables to conclude that there are differences in preferred styles by adolescents by gender; with the male sample showing a greater preference for the adaptive style.

If socio-emotional variables, such as anxiety or the negative interpretation of ambiguous stimuli, which affect the use of adaptive decision-making as highlighted by Bosch et al. (2016), are considered, it is important to design educational programs to train adolescents in decision-making skills. Special attention should be given to the area involving emotions as a previous step to enable adolescents to make more rational decisions. This proposal is especially important in the adolescent stage given that, according to Mann and Friedman (2002), at 15 years of age individuals are cognitively prepared to make decisions with a similar capacity of adults. It is therefore important to offer the relevant experiences that allow them to acquire and practice this skill in order to become fully competent. In this endeavor, formal, non-formal and informal educational settings have an equally important role to play.

Similarly, in order to increase the use of the adaptive decision-making style in adolescent females, it is important to work on aspects such as self-esteem and self-efficacy to help them to perceive themselves as effective decision-makers. Moreover, it is important to make a pedagogical effort in society to improve the perception of females as decision makers.

### Psychological Well-Being and Decision-Making Style

This study has evidenced the existing relationship between psychological well-being and the decision-making style. Thus, adolescents with a higher level of wellbeing show a marked preference for adaptive decision-making strategies. This aspect of subjective wellbeing emphasizes satisfaction with one's own life, development of skills and self-realization.

Along the same lines, the decision-making stages suggested by Byrnes (2002) are directly related to the factors that constitute psychological wellbeing according to Ryff's model. Thus, the stage of establishing objectives and the search for alternatives to achieve them coincides with the purpose in life factor, which assesses people's ability to define their life goals and establish appropriate ways to reach them.

According to the tenets of the Social Cognitive Theory, adolescents suffer from a lack of self-confidence and self-determination. The contents of these two dimensions coincide with the dimensions of *self-acceptance, autonomy*, and *purpose in life* in the variable psychological well-being. In other words, adolescents who have less autonomy, lower self-acceptance and fewer purposes in life use more maladaptive decision-making strategies, such as improvisation.

In contrast, a marked preference for adaptive decision-making strategies is related to greater psychological well-being, which reinforces the value and importance given to peers and the power of the group during this developmental stage.

In order to increase adolescents' subjective perception of their own psychological well-being, we recommend creating programs in secondary schools that include information processing and allow adolescents to activate critical thinking and promote their decision-making skills. Competence-boosting programs would make possible to establish objectives, collate possible alternatives for achieving them, prioritize the alternatives under criteria of importance, and select the best alternative in terms of the decision-making context (Byrnes, 2002). Such programs should be enriched with work modules that enhance adolescents' subjective perception of psychological well-being, which are linked to the dimensions devised by Ryff, and based on the decision-making styles acquired by adolescents.

The importance of this fundamental stage in the development of decision-making skills should be emphasized, along with its relationship with psychological well-being. The formative and educational processes in high schools would benefit from a teaching approach which accompanies adolescents from the very beginning of their secondary stage until they reach the threshold of youth. This opportunity would facilitate the development of decision-making skills linked to cognitive maturation, the development of abstract thought, and environmental mastery (Raleig et al., 2019).

The academic decisions that are taken in the near future are an excellent opportunity to teach students to use an adaptive decision-making style and, consequently, increase their subjective psychological well-being. This perspective, with the support of teachers and family members, and taking into account the challenging identity process of adolescence, could reinforce self-acceptance and the dimensions of subjective well-being. All that promotes greater self-esteem which, in turn, has a direct impact on the use of different decision-making styles (Bethencourt and Cabrera, 2011).

Lastly, the high correlation between decision-making styles and psychological well-being and its dimensions in the two gender samples indicates that educational strategies should be the same for males and females. The results show both gender samples, male and female, value the dimensions with the same degree of importance.

Finally, observing the results of the linear regression analysis, the implication of all the dimensions of psychological well-being in the development of adaptive decision-making strategies in Spanish adolescents can be concluded. Thus, as highlighted in the algorithm that predicts decision-making competence, empowerment of self-acceptance and autonomy, a greater mastery of the environment and the establishment of vital goals implies an increase in the use of adaptive decision-making styles.

These results invite to create an educational intervention with adolescents from various aspects, including formal and non-formal education programs.

This proposal requires an absolute adjustment to the Spanish social and educational model.

On the one hand, the taxonomic works carried out by authors such as Elzo (2016), different categories of adolescents in Spanish society. However, all these categories coincide in attributing to adolescents an interest and concern for their future, being determined to establish goals and objectives. Similarly, the levels of self-esteem and autonomy of Spanish adolescents are, in general, above average. This situation is a very good starting point for the intervention of education professionals. In this sense, the Spanish legislation on regulated education includes the planning of competences, objectives and evaluation criteria for the variables indicated above, from the Early Childhood stage to Baccalaureate.

In addition, this work must be carried out both in the subjects of the common subjects and in the specific subjects that the Spanish curriculum proposes for the education of the personal skills of the students, such is the case of the subject Social Values and civic.

However, the context of non-formal education in Spain is wide and must also be an agent of intervention in the formation of the decision-making competence of adolescents. In this sense, the associative fabric that works with adolescents involved in their educational programs must be promoted, the empowerment of autonomy, self-acceptance, mastery of the environment, the establishment of goals in life and the establishment of positive social relationships such as The way to get Spanish adolescents to use mostly adaptive decision-making strategies.

Finally, if you analyze the variables that protect the most from the use of maladaptive strategies, you can see that it is Autonomy and life parameters. Furthermore, these variables exercise this protective role for both the men's and women's groups, so it is especially important to influence these two dimensions.

## Limitations and Future Research

The limitations of this study indicate the need for a greater differentiation by age range within the concept of adolescence, a broader sample of educational contexts, and greater influence in the programs implemented in education centers in order to enhance the variables.

In this respect, future research possibilities should be addressed to:

Explore the potential relationship of the variables and the social and emotional relationships that adolescents themselves experience within their peer group, the family and/or the educational environment.

Expand the subjective assessment and subjective well-being in different contexts or spheres where adolescents develop.

Explore decision-making based on the influence of social relationships and the regulation of emotions.

Expand on the cognitive processes involved in decision-making skills, the stages of the decision-making process, and the limitations that affect the development of this competence.

## Data Availability Statement

The datasets generated for this study are available on request to the corresponding author.

## Author Contributions

JG-L and FL-N performed the analysis of the state of the matter and drafted the introduction and method. JP-G developed the hypothesis, analyzed the data, and wrote, together with MR-M, the results and discussion. MR-M conducted a general review of the entire document. JG-L helped with the data analysis. All authors read and approved the final manuscript.

## Conflict of Interest

The authors declare that the research was conducted in the absence of any commercial or financial relationships that could be construed as a potential conflict of interest.
